# An Electronic Information Kiosk for Enhancing Patient Accrual for Cancer Clinical Trials: A Pilot and Feasibility Study

**DOI:** 10.7759/cureus.25114

**Published:** 2022-05-18

**Authors:** Morgan D Black, Lilian Esene, Richard McClelland, Heather Mayer, Stephen Welch, Glenn Bauman, Theodore Vandenberg

**Affiliations:** 1 Oncology, London Health Sciences Centre, London, CAN; 2 Medical Oncology, London Regional Cancer Program, Western University, London, CAN; 3 Radiation Oncology, London Regional Cancer Program, Western University, London, CAN; 4 Medical Oncology, London Health Sciences Centre, London, CAN; 5 Medical Oncology, London Regional Cancer Program, Western University, London, CAN

**Keywords:** oncology clinical trials, patient education, accrual, kiosk, clinical trials

## Abstract

Introduction

Low accrual to clinical trials for solid tumors at our institution led to a review of possible modifiable factors within our control. This led to a pilot project to determine whether improved patient awareness could alter accrual rates to active trials.

Methods

An information kiosk was located at the patient library on the ground floor of the London Regional Cancer Program. Adult cancer patients were invited to learn more about clinical trials from our research navigator, including specific trials open in our center, and to participate in the study, which involved a brief satisfaction and demographics survey.

Results

Three hundred and eighty-six (386) patients interacted with the clinical trial information kiosk over the eight weeks it was open. Of these, 32 patients consented and filled out surveys, which indicated an overall positive interaction with the kiosk. Unfortunately, in the time period examined, clinical trial accrual rates appeared to decrease when the pre- and post-kiosk activation periods were compared (44 versus 37 patients accrued to various trials).

Conclusion

Our pilot study found that the implementation of a clinical trial information kiosk was easy to understand and useful for patients to learn more about clinical trials. Barriers to this patient satisfaction translating into increased accrual rates in our center included suboptimal kiosk location and lack of guidance to the kiosk from clerical staff. High patient satisfaction scores support the potential value of permanent clinical trial information kiosks in our cancer center, but this requires increased attention to visibility, location, and staff education.

## Introduction

Background

The National Cancer Institute (NCI) describes clinical trials as “…essential for moving new methods of preventing, diagnosing, and treating diseases from the laboratory to physicians' offices and other clinical settings and, ultimately, to improve care and quality of life”. A major barrier to the successful completion of clinical trials is patient accrual. Accrual to clinical trials in oncology is often described as challenging since it is a complex, multistep process with many potential barriers. Low patient accrual rates have been found to contribute to higher institutional costs and extended periods of enrollment [1] to impede the translation of preclinical research. This prevents clinical trials from meeting their initial goals and hinders the interpretation of results [2,3]. Contemporary clinical trials have excluded many patients, resulting in suboptimal generalization of results [4]. A review of 138 radiation oncology registered randomized trial failures noted an increasing risk of accrual failure in more recent trials [5].

In an attempt to better understand the issue of slow accrual, the NCI and American Society of Clinical Oncology (ASCO) convened a group of experts to examine the challenges to patient enrollment in clinical trials. This group found barriers in three areas, including the site/organizational level, the physician/provider level [6], and the patient/community level [7]. The physician/provider level included a willingness to refer a patient to a study, lack of knowledge about open clinical trials, and concerns over a patient's ability to participate in a trial. The final barrier, at the patient/community level, included patients being unaware of clinical trials and the stringencies of the protocols [8].

In addition, a more recent study performed at MD Anderson Cancer Center looked at 30+ years of clinical trial experience in an attempt to identify weaknesses of current clinical trial models in an effort to improve trial design and accrual in the future. From January 1981 to March 2011, a total of 145,214 participants were enrolled in 4,269 phase I-III clinical trials, with a median enrollment of 16 and an average rate of 8.7 participants enrolled per year [2]. Eighteen percent of trials were categorized as slow accruing with these trials having strong associations with national cooperative group trials, time from trial activation to first enrollment, and maximum targeted accrual. They also found that trials requiring 70+ days between activation and the first participant enrolled had a higher chance of slow accrual throughout the trial [2]. A recent Canadian survey of primarily medical and radiation oncologists [9] revealed time constraints, including increased paperwork, requirements for patient education and extra clinic visits, and lengthy follow-up as barriers to accrual.

These studies have helped clinicians and researchers to better understand the reasons behind slow patient enrollment in clinical trials. To address how to improve trial accrual, a group of researchers out of the MD Anderson Cancer Center sought to analyze factors affecting successful clinical trial enrollment. They found that enrolled trial participants were more likely to have been presented information at earlier appointments or earlier in their course of treatment, with the earlier appointment intervention to be the strongest predictor of enrollment [10].

In view of the above observations, concerns were raised regarding slow patient accrual for many of our clinical trials for solid tumors at our institution. Even though there were 84 open clinical trials at the London Regional Cancer Program (LRCP), patient accrual remained low. This was despite the clinical practice occurring within an academic program and the use of posted flow charts of currently-available trials and eligibility in clinics for each disease site. Reasons for slow patient accrual, we felt, were likely due to a combination of lack of patient awareness of trials, lack of time to discuss clinical trial options in busy clinics, stringent eligibility criteria, and lack of funding to employ clinical research staff to manually review clinic schedules for possible candidates.

Our single-center experience, as well as previous studies, have found that many patients are interested in clinical trials if explained to them in an appropriate manner early in their treatment journey and when they understand basic trial concepts such as informed consent, independent ethics approval, freedom to withdraw from treatment without impact on standard care, etc. We proposed that a clinical trial information kiosk would help solve issues of time and funding as most patients have ample amounts of time while waiting for their appointment, and economies of scale will improve efficiencies and reduce cost per patient.

Rationale

Previous groups have identified a major barrier to clinical trial accrual as poor awareness of existing trials. With the background knowledge that earlier intervention and presentation of clinical trial information have the potential to increase trial accrual [10,11], we proposed a clinical trial information kiosk for patients to explore at their oncology appointments. We targeted the top clinical trials ranked by the multi-disciplinary disease site teams for each main tumor site for significant enrollment potential.

Potential benefits to increased accrual include fewer studies closed due to poor accrual, increased financial success for our center's Clinical Cancer Research Unit (CCRU), increased success in negotiating collaboration in academically and clinically important trials, and increased publications in peer-reviewed academic journals. For patients, benefits include increased knowledge of clinical trials relevant to their condition and more treatment options.

Study objectives

Our primary objective was to quantify the clinical trial accrual rates in a subset of trials that are selected as priority trials and were open both two months before and at least three months after implementation of the clinical trial information kiosk. Accrual rates before and after implementation were compared and included patients who accessed the kiosk within the two-month period but may not have consented to a trial until a month after that period, as long as the trial consented to was one the patient addressed as a result of a kiosk interaction. The assumption was that patients required a month from exposure to the kiosk to be placed on a trial, so the extra month was to account for delayed effects.

Our secondary objectives were 1) to determine the proportion of patients who accessed the kiosk provided information so that they could be contacted by the research navigator, 2) to determine what proportion of patients who were contacted the research navigator were eligible to participate in a clinical trial, 3) to determine what proportion of patients eligible for a clinical trial actually participated, 4) to evaluate patient satisfaction with the clinical trial information kiosk, and finally, 5) to determine the number of individuals who interacted with the information kiosk compared to the total number of cancer patients seen in clinic when the kiosk is open. Our exploratory objective was to examine the demographic correlations of patients seeking additional information.

## Materials and methods

Study population

All cancer patients with non-hematologic malignancies with a clinic appointment and/or treatment at the LRCP were eligible for participation in this feasibility study. To participate in the study, patients had to be 18 years of age or older and diagnosed with a solid tumor cancer (including breast, central nervous system, gynecologic, gastrointestinal, genitourinary, head and neck, liver, melanoma, neuroendocrine, sarcoma, thoracic, thyroid). Additionally, patients had to be able to provide independent informed consent. Patients were excluded from the study if they were less than 18 years of age, unable to provide informed consent, and unable to read and comprehend written English.

Approximately 250 cancer patients are seen daily in the LRCP radiation and medical oncology clinics weekly. We aimed to recruit over a two-week period 125 patients (5% of the total 2,500 patients seen in two weeks at LRCP) through signage, a handout given to patients upon check-in as well as a volunteer guide. We then aimed to run the kiosk for an additional seven weeks, thus anticipating approximately 560 patients interacting with the information kiosk.

Study methodology

Ethics approval was obtained from Western University Health Sciences Research Ethics Board (REB#: 111988). Written informed consent was obtained from each participant prior to interaction with the clinical trial information kiosk. The information kiosk was located at the patient library on the ground floor near the radiation treatment facilities. This kiosk was outfitted with an iPad and signage briefly outlining the title and purpose of the kiosk. Patients were made aware of the kiosk in several ways. We designed a four by eight-foot kiosk banner that was eye-catching and humorous to attract initial attention so that patients would be more likely to meet with the attendant who would facilitate further discussion. An introductory card was to be given at reception desks to patients/families outlining the existence of the kiosk. A second identical banner was located on the second-floor entrance where clinics are located and systemic therapy is delivered. At the kiosk, patients could indicate their interest in participating in a trial, obtain applicable basic trial information, and sign a paper or electronic consent form granting a clinical research associate access to their electronic health record to contact them regarding potential trials they may be eligible for.

Once consented, the kiosk attendant described the clinical trial information kiosk along with the possible benefits that might be associated with participation in this study, including further clinical trial information as well as a potential invitation to explore clinical trials further with the research navigator to review their request for further information, contact them with potential options and contact their oncologist(s) to discuss their potential enrollment in a specific trial. The pilot kiosk utilized general information available to the public from the Stanford Cancer Institute Clinical Trials website [12]. A Canadian site was also used [13]. They would then be asked to review a list of possible trials (trial names are presented in Appendix 1) they could be eligible for based on their cancer diagnosis (e.g., breast, colorectal, lung, etc.; Appendix 2). This kiosk included information on the top trials for each disease site chosen by the cancer disease site teams based on trials open for at least five months and likely to accrue three patients over six months for major accruing tumor sites and at least one patient over six months for other sites. The main exclusion criteria for trials included at the kiosk would be trials that would be imminently closing in less than three months after the kiosk was activated as well as trials that were highly selective and unlikely to find an eligible patient in the brief time period available for the study. Additionally, the number of trials included was limited in order to not overwhelm the human resources available. Finally, patients had the option of having any trial information they might be interested in sent to their home or their email address or obtain a paper copy of the information.

Participants were asked to complete satisfaction and demographic survey and were allowed to proceed through the survey if they refused to answer a question. After a two-week run-in phase, accrual, logistical, and communications issues were reviewed to ensure the kiosk project was functioning efficiently and to review patient satisfaction.

Data collection

Each consented patient was assigned a unique study ID number. All participant responses were de-identified and linked to the ID number. Data was collected on the number of patients visiting the kiosk as well as on the patients who agreed to participate in the kiosk survey and have demographic information collected, which included age, sex, urban versus rural using the Ontario government definition of rurality based on community population of less than 30,000 or living more than 30 minutes travel time from such a community, highest educational level, occupation, caring status, occupational status and income. Participants were also asked: "Since your diagnosis of cancer, has anyone discussed with you whether you would like to take part in cancer research? If yes, did you go on to take part in cancer research?" [14]. We obtained baseline information by examining the number of patients who interacted with the kiosk as a proportion of total patients seen in clinics on active kiosk days. Any issues in the trial algorithm information handouts that were identified in the patient satisfaction surveys were identified. Additionally, we investigated issues the kiosk attendant experienced (e.g., difficult questions, insufficient paper materials, overwhelmed feelings).

Statistical analysis

We examined clinical trial accrual rates for the entire period, including the first two weeks' run-in phase. In both the run-in and main phases, we examined patient overall accrual rates and accrual rates in cancer clinical trials by comparing rates before and after implementation of the kiosk. Clinical trial accrual was measured by the ratio of actual to projected patient accrual over the length of the trial, accrual in a two-month period before activation of the pilot, and a subsequent two-month period starting one month after activation of the kiosk. For the purposes of this initial pilot, we deemed a clinical trials accrual increase of greater than 30% for trials included at the kiosk to be a significant increase in cancer trial accrual after a nine-week period where we estimate that about 560 patients will have accessed the kiosk.

Patient satisfaction with the clinical trial information kiosk was qualitatively assessed using a modified “Was It Worth It (WIWI)” satisfaction assessment (Appendix 3) [15]. This tool determines the proportion of patients reporting worthwhile participation [16]. Descriptive characteristics of the study population were analyzed using standard statistics, including proportion and percentages for categorical variables and mean and standard deviations for continuous variables. For our exploratory objective, univariate analysis and logistic regression modeling were performed on patient demographic and treatment characteristics to ascertain predictors of satisfaction [17].

## Results

**Figure 1:**
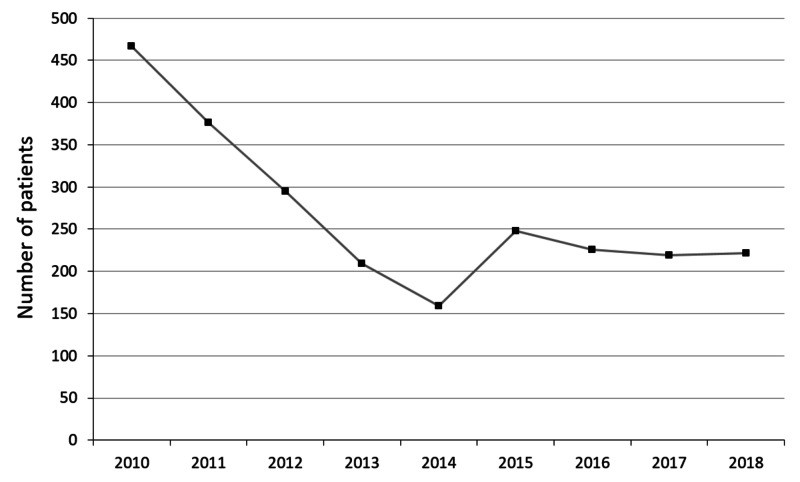
Clinical trial accrual by year at London Regional Cancer Program Clinical Cancer Research Unit

Total accrual over the last five years at our cancer center is outlined in Figure 1.

It was initially predicted that approximately 5% (488) of patient visits in our cancer center would interact with the kiosk (39 days*250 patient visits per day=9,750 total patient visits; 9,750 patient visits*0.05=488). The trial opened on July 9, 2018, a week later than anticipated, and continued for eight weeks. Three-hundred and eighty-six (386) patients visited the kiosk over the 39 days the kiosk was open (eight weeks minus one statutory holiday), which was 79% (386/488) of the target goal when opening the kiosk.

This was affected by a delayed start due to logistical and administrative issues. Also, visual cues (i.e., balloons) had to be taken down due to potential interference with the cancer center’s ventilation system. However, the last month of accrual matched the goal of interaction with 12 patients, on average, daily.

Of the 18 clinical trials featured at the kiosk (Appendix 2), ten clinical trials were available through the Canadian Cooperative Clinical Trials Group (CCTG) and nine were industry-sponsored. Eight trials were adjuvant (three breast, one each of colon, renal cell, head and neck, esophagus, and non-small cell lung).

Thirty-two (32) patients consented and filled out surveys, 75% of whom indicated that they had not been asked by their cancer care team whether they were interested in participating in clinical trials research (Table 1). It should be noted that even with the best of intentions, the care environment is not conducive to assessing the eligibility of patients for clinical trials nor discussing trials due to the high-pressure nature of the multi-tasking clinical environment.

**Table 1:** Demographics characteristics of study participants

	Survey respondents, n (%)
Median age	60.8 years (SD=13.4)
Sex (n=32)	
Male	14 (44)
Female	18 (56)
Disease site (n=27)	
Hematological	9 (33)
Breast	4 (15)
Gastrointestinal	3 (11)
Gynecological	1 (4)
Lung	1 (4)
Genitourinary	1 (4)
Unknown	8 (30)
Geographic location (n=23)	
Urban	18 (78)
Rural	5 (22)
Highest education level (n=31)	
High school	8 (26)
College	8 (26)
University	13 (42)
Graduate	2 (6)
Occupation (n=29)	
Agriculture or forestry or fishery worker	1 (3)
Crafts and trades	2 (7)
Factory worker or technician	3 (10)
Manager	5 (17)
Professional	6 (21)
Services or sales worker	5 (17)
Nurse	2 (7)
Teacher or professor	2 (7)
Occupational Status (n=32)	
Part-time (<37.5 hr/week)	4 (13)
Full-time (>37.5 hr/week)	4 (13)
Retired	15 (47)
Unable to work	9 (28)
Income (n=22)	
Less than $20,000	2 (9)
$20,000 to $34,999	6 (27)
$35,000 to $49,999	3 (14)
$50,000 to $74,999	2 (9)
$75,000 to $99,999	6 (27)
$100,000 to $149,999	2 (9)
Over $150,000	1 (5)
Since your diagnosis of cancer, has anyone discussed with you whether you would like to take part in cancer research? (n=32)	
No	24 (75)
Yes	8 (25)

Twenty (20) solid tumor patients and nine hematology-oncology patients filled out questionnaires allowing research staff to access the electronic health record (EHR). Of the solid tumor patients, 17 were not eligible for an active clinical trial, three were eligible, two agreed to be interviewed for the Ontario-wide Cancer Targeted Nucleic Acid Evaluation (OCTANE) study but are responding to standard treatment so are not yet ready to start investigational therapy, and one has agreed to participate in the MAC.19 trial but was not randomized until four months after the pilot was closed. 

The demographic characteristics of the 32 participants are outlined in Table 1. Not all participants elected to respond to each demographic question thus changing the total number of participants for each characteristic. The median age was 60.8 years with a standard deviation of 13.4 years. Fourteen males and 18 females completed surveys. Five (22%) resided in rural residences while 18 (78%) were from urban centers. Twenty-three of 31 participants (74%) had a college or university education. Only 25% (8/32) of participants replied 'yes' to the question "Since your diagnosis of cancer, has anyone discussed with you whether you would like to take part in cancer research?".

Patient satisfaction survey scores are outlined in Table 2 based on a seven-point Likert scale.

**Table 2:** Mean scores for patient satisfaction * On a seven-point Likert scale

Question	Mean score*
Did you find the Clinical Trial Information kiosk useful?	6.2
Was the information easy to understand?	6.5
Are you interested in getting more information about a clinical trial based on your interaction at the kiosk?	6.6
Would you recommend the clinical trial information kiosk to other cancer patients?	6.2

Overall, participants found the information kiosk useful and easy to understand. Additionally, the majority of participants were interested in obtaining more information about clinical trials based on their interaction with the kiosk and would recommend the kiosk to other cancer patients. Free comments included "more direction to the kiosk would be good" as well as "signage for the kiosk could be better".

With respect to the primary outcome of interest, accrual rates in the last two months before kiosk activation and two months starting a month after activation were compared (Table 3).

**Table 3:** Monthly clinical trial accrual from March 1, 2018 to December 31, 2018 CNS - central nervous system; GYN - gynecologic; GI - gastrointestinal; GU - genitourinary; H&N - head and neck; Mel - melanoma

Site	Trial	Mar	Apr	May	Jun	Jul	Aug	Sep	Oct	Nov	Dec
Breast	DUCHESS	1	3	0	0	0	2	2	0	2	1
	MAC.19	0	0	0	1	0	0	0	0	0	2
	MAC.20	0	0	1	0	1	0	0	1	1	0
CNS	TOCA5	1	1	0	0	0	0	0	0	0	0
GYN	C31004	0	0	0	0	0	0	0	0	0	0
	EMPOWER	0	0	0	0	0	0	0	0	0	0
GI	CO.21	1	0	0	0	0	1	0	0	0	0
	CO.27	0	0	0	0	0	0	0	0	0	0
	PA.7(excluded)	0	1	1	1	1	0	0	0	0	0
GU	CHECKMATE901	0	0	1	0	0	0	0	0	0	0
	IND.234	0	0	0	0	0	1	3	0	1	0
	KEYNOTE564	1	0	0	0	0	0	0	1	1	0
H&N	IMVOKE	0	0	0	0	0	0	0	0	0	0
Mel	ME.13	1	1	0	1	1	1	1	0	3	0
Thoracic	BR.31	0	0	0	0	0	0	0	0	0	0
	CHECKMATE577	0	0	0	0	0	0	0	0	0	0
	IND.227	0	0	1	1	0	0	0	0	0	0
Multiple	CAPTUR PM1	0	0	0	1	4	2	0	3	0	0
	OCTANE	9	7	16	21	9	12	12	16	10	13
TOTAL		14	12	19	25	15	19	18	21	18	16

Total accrual pre- and post-activation of the kiosk was 44 versus 37 patients. One study (PA.7) closed to accrual earlier than expected and was not counted. There was no accrual to gynecologic or head and neck sites. Accrual by tumor site was breast (two versus four), GI (zero versus one), melanoma (one versus two), genitourinary (one versus four), thoracic (two versus zero), and multiple sites (38 versus 26). Multiple sites included a study to assess if a malignancy had a molecular profile that would allow entry into a basket of subsequent phase 2 trials for any solid tumor (37 versus 24 and one versus two, respectively).

Barriers to effective accrual included a suboptimal location in relation to traffic flow; the kiosk initially had vague signage and was located next to a coffee shop and looked like an extension of the coffee shop area. Clerical staff at the clinic reception areas were too busy to routinely give out tickets reminding patients of the kiosk. This was rectified by improved signage identifying the kiosk, additional signage on the more heavily trafficked second floor, and periodic walkabouts by the kiosk attendant.

## Discussion

Accrual to clinical trials after the kiosk intervention was less than expected. There are several possible explanations, including that the kiosk implementation occurred during the summer months, and there was an unexpected sudden shortage of clinical trial staff, which may have delayed accrual. Some patients did not wish to go from the main clinic area on the second floor to the ground floor despite an elevator being available. A shortage of space precluded the placement of the kiosk in the main clinic area. Despite the signage, the kiosk was easily confused with an extension of the coffee shop seating area. Additionally, clinical trial eligibility restrictions likely played a role. Trial accrual was dominated by the OCTANE study, which was more likely driven by physicians rather than patients.

We were surprised that there was no effect of the kiosk on trials for patients with early cancers focusing on exercise to prevent recurrence (MAC.20 and CO.21). Overall accrual to CCTG trials in the planned analysis of the April - June period compared to August - October was 16 versus 19 participants. It is possible that there is a delayed effect on trial accrual that exceeds the assumed time frame of the pilot. One possibility for the less than expected effect on clinical trial accrual could have been because our patient population is typically more rural-based and elderly. Rural patients have logistical challenges accommodating increased travel for more visits associated with trials, and an elderly population with more comorbidities may well not be as interested in pursuing clinical trials. The cancer center included in this study should not be compared to a large academic center more focused on research as our mandate is much broader. 

A sensitivity analysis incorporating five months from March to July versus from August to December showed 84 versus 89 accruals. Removing the OCTANE study (but including its related study, Canadian Profiling and Targeted Agent Utilization Trial [CAPTUR]) patients - 23 versus 27 patients were accrued. More relevant is the number of patients who consented to allow access of CCRU staff to their EHR who presumably had more detailed information about trials and increased interest went on to participate in a clinical trial. One of these 20 patients was randomized to an adjuvant breast trial. It is possible we may be missing a longer delayed effect for some trials, especially for some of the adjuvant ones that allow accrual up to a year after diagnosis.

The kiosk visits approached expectations toward the end of the period but were less than hoped for (~79%). The kiosk intervention started conversations with 386 patients, raising awareness for future discussions with oncologists in their cancer journey. Of the patients that consented to participate in the study, high satisfaction scores (>6 out of 7) for the kiosk were given, suggesting that the service was helpful. It is unclear whether a longer study period, both for the time the kiosk is open or a longer follow-up for each patient, would have altered these results. Greater attention to visibility, location and accessibility, and staff education to encourage kiosk use should be addressed.

## Conclusions

This pilot study found that accrual to cancer clinical trials was not appreciably increased through the use of an information kiosk. Additionally, the lack of signage and kiosk location resulted in a lower than expected number of patients interacting with the clinical trial kiosk. Optimizing the kiosk experience and increasing patient interactions with the kiosk in the future would require additional signage, improved location, and further education to clinical staff to direct patients to the kiosk. However, further research is required to justify this concept. Despite accrual to clinical trials not being observed to increase significantly with the implementation of the information kiosk, the concept was overwhelmingly supported by cancer patients who found it useful and easy to understand.

## Figures and Tables

**Figure 2:**
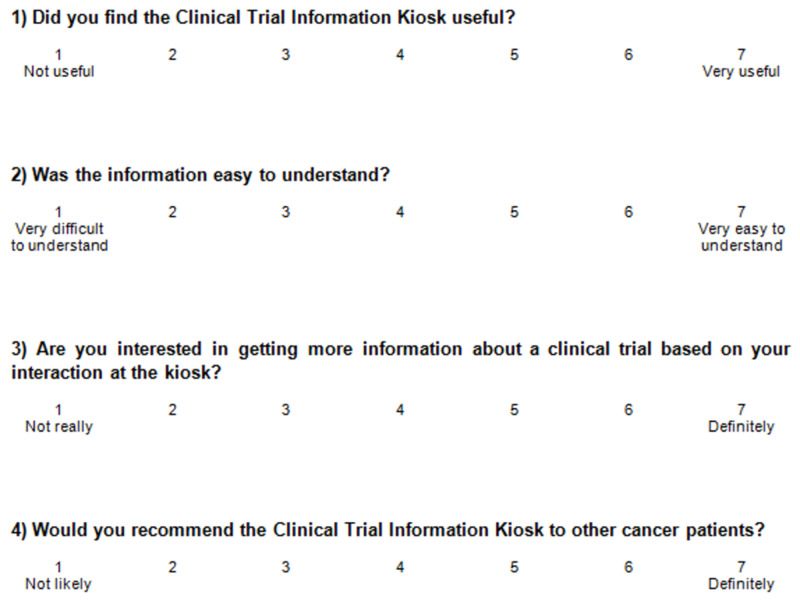
Patient satisfaction survey

## Appendices

Appendix 1

DUCHESS - Evaluation of the DCIS Score for Decisions on Radiotherapy in Patients With Low/Intermediate Risk DCIS

MAC.19 - A Randomized Phase III Trial Evalulating the Role of Axillary Lymph Node Dissection in Breast Cancer Patients (cT1 -3 N1) Who Have Positive Sentinel Lymph Node Disease After Neoadjuvant Chemotherapy

MAC.20 - Randomized Phase III Trial Evaluating the Role of Weight Loss In Adjuvant Treatment of Overweight and Obese Women with Early Breast Cancer

TOCA5 - Toca 511 & Toca FC Versus Standard of Care in Patients With Recurrent High Grade Glioma

C31004 - A Study of Sapanisertib, Combination of Sapanisertib With MLN1117, Paclitaxel and Combination of Sapanisertib With Paclitaxel in Women With Endometrial Cancer

EMPOWER - The EMPOWER Study: Endometriosis Diagnosis Using microRNA

CO.21 - A Phase III Study of the Impact of a Physical Activity Program on Disease-Free Survival in Patients with High Risk Stage II or Stage III Colon Cancer: A Randomized Controlled Trial (CHALLENGE)

CO.27 - A Phase III, Randomised, International Trial Comparing mFOLFIRINOX Triplet Chemotherapy to mFOLFOX for High Risk Stage III Colon Cancer in Adjuvant Setting

PA.7 - A Randomized Phase II Trial of Gemcitabine and Nab-Paclitaxel vs Gemcitabine, Nab-Paclitaxel, Durvalumab and Tremelimumab as 1st Line Therapy in Metastatic Pancreatic Adenocarcinoma

CHECKMATE901 - Study of Nivolumab in Combination With Ipilimumab or Standard of Care Chemotherapy Compared to the Standard of Care Chemotherapy Alone in Treatment of Participants With Untreated Inoperable or Metastatic Urothelial Cancer

IND.234 - Prostate Cancer Biomarker Enrichment and Treatment Selection

KEYNOTE564 - Safety and Efficacy Study of Pembrolizumab (MK-3475) as Monotherapy in the Adjuvant Treatment of Renal Cell Carcinoma Post Nephrectomy

IMVOKE - A Study of Atezolizumab (Anti-Pd-L1 Antibody) as Adjuvant Therapy After Definitive Local Therapy in Patients With High-Risk Locally Advanced Squamous Cell Carcinoma of the Head and Neck

ME.13 - A Randomized Phase III Study of Duration of Anti-PD-1 Therapy in Metastatic Melanoma (STOP-GAP)

BR.31 - Double Blind Placebo Controlled Controlled Study of Adjuvant MEDI4736 In Completely Resected NSCLC

CHECKMATE577 - An Investigational Immuno-therapy Study of Nivolumab or Placebo in Participants With Resected Esophageal or Gastroesophageal Junction Cancer

IND.227 - Pembrolizumab in Patients With Advanced Malignant Pleural Mesothelioma

CAPTUR PM1 = Canadian Profiling and Targeted Agent Utilization Trial

OCTANE - Ontario-wide Cancer Targeted Nucleic Acid Evaluation

Appendix 2

**Table 4:** Clinical trials featured at the clinical trial information kiosk at LRCP A - adjuvant; M - metastatic/recurrent; LRCP - London Regional Cancer Program

Disease site	Clinical trials		
Breast	MAC.20^A^	MAC.19^A^	DUCHESS^A^
Central nervous system	TOCA5^M^ (glioblastoma)		
Gynecologic	C31004^M^ (endometrium)	EMPOWER^M^ (cervix)	
Gastrointestinal	PA.7^M^ (pancreas)	CO.21^A^ (colon)	CO.27^M^ (colon)
Genitourinary	Keynote564^A^ (renal cell)	Checkmate 901^M^ (urothelial)	IND234^M^ (prostate)
Head and neck	ImVoke^A^		
Melanoma	ME.13^M^		
Thoracic	Checkmate 577^A^ (esophagus)	BR.31^A^ (NSC lung)	IND227^M^ (mesothelioma)
Multiple sites	OCTANE/CAPTUR^M^		

 Appendix 3
